# Long-term prognosis analysis of PARACHUTE device implantation in patients with ischemic heart failure: a single-center experience of Chinese patients

**DOI:** 10.1186/s13019-021-01484-0

**Published:** 2021-04-20

**Authors:** Jianghua Li, Huadong Liu, Qiyun Liu, Cheng Liu, Wei Xiong, Wei Ma, Baowei Zhang, Shaohong Dong, Tangzhiming Li

**Affiliations:** 1 Department of Cardiology, Shenzhen Cardiovascular Minimally Invasive Medical Engineering Technology Research and Development Center, Shenzhen People’s Hospital, Shenzhen, 518020 Guangdong China; 2 The Second Clinical Medical College, Jinan University; The First Affiliated Hospital, Southern University of Science and Technology, Shenzhen, 518020 Guangdong China; 3 Department of Cardiovascular Disease, Peking University First Hospital, Beijing, 100034 Beijing China; 4 Department of Cardiology, Shenzhen People’s Hospital, 1017 Dongmen North Road, Shenzhen, Guangdong China

**Keywords:** Myocardial infarction, Left ventricular systolic function, Heart failure, Mortality

## Abstract

**Background:**

Heart failure (HF) is one of the leading causes of mortality and morbidity. The PARACHUTE device is designed to partition for left ventricular (LV) apical aneurysm post extensive anterior myocardial infarction (MI). However, the long-term prognosis of the PARACHUTE device post-implantation is unclear.

**Methods:**

From November 2015 to April 2017, six subjects with New York Heart Association Classes II, III and IV ischemic HF, LV ejection fraction between (LVEF) 15 and 40%; and LV anterior apical aneurysm were enrolled in our center. The cumulative event rates for MI, hospitalization, and mortality were documented. Further assessment of LVEF, LV end-diastolic diameter (LVEDD), and estimated pulmonary artery pressure were determined by echocardiography core laboratory. For quantitative data comparison, paired *t*-test was employed.

**Results:**

Device implantation was successful in all six enrolled subjects, and acute device association adverse events were not observed. At 4.6 ± 1.7 years follow-up, major adverse cardiac events (MACEs) were found in 50% patients, and the survival rate was 86.7%. We observed that the LVEF was significantly elevated after deployment (46.00 ± 6.00% vs. 35.83 ± 1.47%, *P* = 0.009). Besides, the LVEDD elevated after MI (51.17 ± 3.71 vs. 62.83 ± 3.25, *P* < 0.001) was revealed, but the device sustained preserved LVEDD after implantation.

**Conclusion:**

The PARACHUTE device is an alternative therapy for patients with severe LV maladaptive remodeling. However, the device seems to increase the HF ratio.

**Trial registration:**

NCT02240940

**Supplementary Information:**

The online version contains supplementary material available at 10.1186/s13019-021-01484-0.

## Introduction

Left ventricle (LV) maladaptive remodeling after acute myocardial infarction (MI) has been well documented in experimental and clinical trials [1, 2]. During the past decades, the number of hospitalizations for acute MIs has increased significantly. Despite the treatment improvement in MI, the long-term prognosis of MI-related LV remodeling remains unfavorable.

The concept of percutaneous ventricular restoration (PVR) therapy of dilated LV is based on the premise of a dedicated partitioning device for LV volume reduction and geometric reconfiguration [3–5]. PARACHUTE® (CardioKinetix, Redwood City, CA, USA) is the first device designed for PVR, and it minimizes the risk of other invasive methods.

Previous clinical trials implied that the PARACHUTE device may reduce cardiac dimensions and end-diastolic wall stress, thereby benefitting patients with LV dilation post-MI. However, little is known about the long-term cardiac function, life quality, and major adverse cardiac events (MACEs) of PARACHUTE-implanted patients.

Therefore, we retrospectively collected the clinical and average 4.6 years follow-up data to analyze the PARACHUTE device-related outcomes and complications, and we also performed a per- versu post-implantation pairwise comparison of patients’ echocardiography parameter. In addition, we investigated the potential factor of an adverse event in these patients.

## Methods

### Study design and patient selection criteria

The Percutaneous Ventricular Restoration in Chronic Heart Failure due to Ischemic Heart Disease (PARACHUTE) China (NCT02240940, https://clinicaltrials.gov/ct2/show/NCT02240940) is a prospective, non-randomized observational study designed to assess the safety and efficacy of the PARACHUTE device. Clinical and echocardiographic follow-up was performed.

Inclusion criteria were as follows: enrolled cases were patients with symptomatic ischemic HF with NYHA Classes II, III, and IV. These patients were 18–75 years old with LV motion abnormalities secondary to anterior MI. LVEF ranged from 15 to 40%, which was measured by the 2D echocardiography core laboratory (core lab). All participants with myocardial ischemia underwent revascularization and received optimal and standard HF medical therapy for at least 3 months before enrollment.

The exclusion criteria were as follows: significant valvular stenosis or regurgitation, chronic obstructive pulmonary disease, end-stage renal disease requiring hemodialysis, cerebral vascular accident, or transient ischemic attacks occurring within 6 months. Selected computed tomography (CT) was applied for proper device size selection. All sites obtained approval from the Ethics Committee before the study began, and written informed consent was obtained for all patients at the appropriate time before involvement in this study. After the implantation of the device, clinical and echocardiographic follow-up was performed annually for up to 3 years.

### Study device and procedure

Details of the device and procedure had been previously published [3–5]. In brief, the procedure was performed in a catheterization laboratory with the patients usually under conscious sedation. Multi-slice CT (Supplementary Figure 1) was implemented to provide accurate measurements and rule out LV apical thrombus, pseudochorda, or severe calcification. After the expansion of the device, the occlusive membrane provides a barrier to seal off the static chamber on the distal side of the device. All patients received low-dose aspirin and anticoagulation with warfarin for at least 12 months of post-device implantation.

### Transesophageal echocardiography

Patients underwent 3D-transthoracic echocardiography (TTE) and 2D-TEE with IE-33 Philips systems (Andover MA, USA) by using a 3D X5–1 PureWave matrix-array transducer. Images were digitally acquired for off-line reconstruction (for 3D-TEE images) and interpretation. 2D-TEE was used from basal to mid-esophageal levels.

### Date collection and follow-up

All study-related data were collected on standardized case report forms [3–5] We assessed the successful delivery and deployment of the PARACHUTE device. The average of 4.6 years of follow-up data on MACEs was recorded. MACEs were defined as cardiac death, emergent cardiac surgery, cardiac tamponade, peripheral embolization, new or worsening HF, endocarditis or device infection, device migration or embolization. We collected echocardiography measurements, including LV end diastolic diameter (LVEDD), LLVEF, and estimated pulmonary artery pressure (PAP). Functional parameters such as NYHA functional class ranking were also determined. Data management was performed by contract research organization, and adverse events were adjudicated by an independent clinical events committee.

### Statistical design and statistical analysis

The key efficacy evaluation was based on transthoracic TTE analysis from baseline to device implantation, including the follow-up data. The MACEs and efficacy analyses were performed on those who were discharged from the hospital after being treated with the study device. The following baseline characteristics were summarized using the mean ± standard deviation (SD) for continuous variables and counts and percentages for categorical variables. Paired *t*-test was conducted to compare the quantitative data; the signed-rank test was applied for the ranked data comparison. All statistical tests were performed using a two-sided test; *P* ≤ 0.05 was considered statistically significant. All analyses were performed using SPSS 21.0 (USA).

## Results

### Patient characteristics

**Fig. 1:**
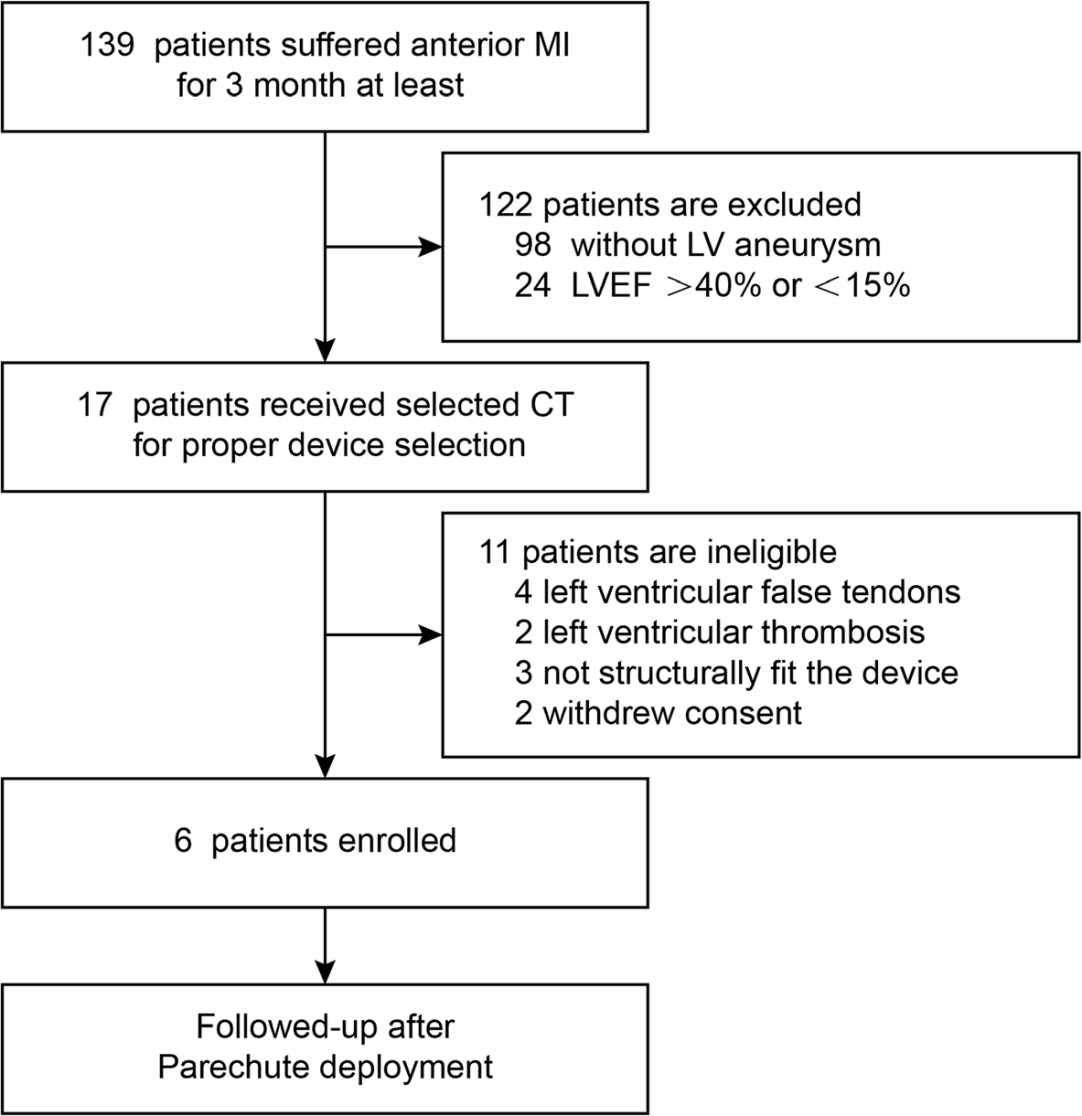
Disposition of subjects enrolled. Six post-myocardial infarction eligible subjects with aneurysm received the PARACHUTE device

From November 2015 to April 2017, 139 patients with post-anterior MI were initially enrolled in our trial (Fig. 1). These patients were screened by 2D TEE and Doppler examinations. A total of 122 of these patients were ineligible, 98 subjects had not developed an aneurysm in the LV, and 24 patients had an LVEF less than 15% or over 40%. Device sizing and anatomical approval were granted by the central CT core lab. Seventeen patients received CT scan for proper PARACHUTE device selection; 11 were excluded for LV false tendons (four subjects), LV thrombosis (two subjects), and not being structurally fit for the device (three subjects); two patients withdrew their consent. Six patients were discharged with the PARACHUTE device and followed up for cardiac function and clinical outcomes. The mean follow-up time was 4.6 ± 1.7 years.

All the patients must have had a diagnosis of NYHA Class II, III, or IV before enrollment to be included in this study. Ischemic heart disease was present in all patients. All subjects with LV descending artery MI had a prior percutaneous coronary intervention. The average age of the patients was 55.7 ± 11.3 years old, the average time from an acute MI to PARACHUTE implantation was 13.5 ± 12.9 months, and BMI was 26.0 ± 2.0. Three patients had single-vessel occlusion, the rest had multivessel disease, and all revascularization was complete in their first hospitalization (data not shown). Five males and one female were involved. We hospitalized one patient for HF within 12 months. The baseline demographic data are shown in Table 1. All patients had LV post-MI aneurysm, and no patient received ICDs or CRTs.

**Table 1:** Baseline demographic data

Characteristics	Value
Age (Years)	55.7 ± 11.3
Male n, (%)	5 (84.3%)
BMI	26.0 ± 2.0
Previous medical history	
Hypertension n, (%)	5 (66.7%)
Hyperlipidemia n, (%)	1 (16.7%)
Diabetes n, (%)	1 (16.7%)
Stroke n, (%)	0 (0%)
Smoking n, (%)	5 (66.7%)
Recent rehospitalization of HF n, (%)	1 (16.7%)
Time form previous MI (month)	13.5 ± 12.9
Prior ICD implantation	0 (0%)
Prior CRT device	0 (0%)
Prior PCI	6 (100%)
Prior CABG surgery	0 (0%)
LV wall motion types	
Akinetic	0 (0%)
Dyskinetic	0 (0%)
Aneurysm	6 (100%)

*Abbreviation*: *BMI* body mass Index, *HF* heart failure, *MI* myocardial infarction, *ICD* implantable cardioverter defibrillators, *CRT* cardiac resynchronization therapy, *PCI* percutaneous coronary intervention, *CABG* coronary artery bypass grafting, *LV* left ventricular

**Fig. 2:**
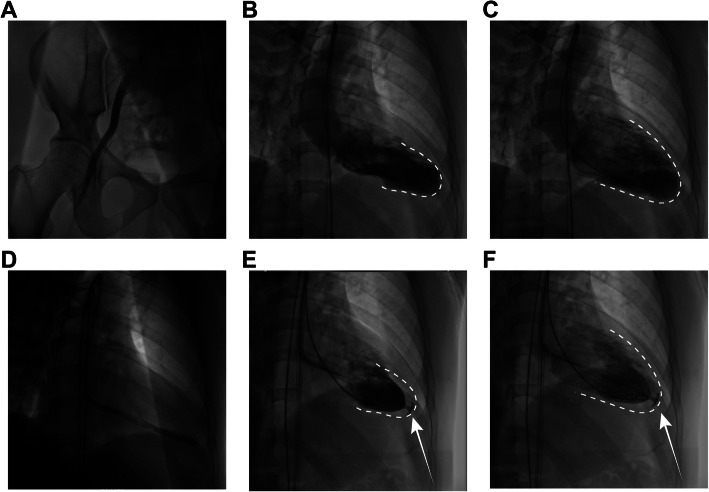
Sequence of the PARACHUTE device implantation in the left ventricle. The operation was performed through the femoral artery (**a**). LV angiography was executed to observe the systolic (**b**) and diastolic (**c**) LV geometry. The device was placed in contact within the LV apical wall (**d**). After balloon inflation to facilitate device expansion, the LV geometric reconfiguration was evaluated in the systolic (**e**) and diastolic (**f**) phases. The white dotted line indicates the outline of the chamber morphological characteristics before the device implantation. The white arrow indicates the implanted PARACHUTE device

As presented in Table 2, the PARACHUTE devices were implanted through the femoral artery of all the six subjects (Fig. 2a). The procedure was successful in six (100%) patients, without a perforation case. No major vascular complications were found, and no PARACHUTE device was expanded or deployed improperly. In addition, no acute thrombosis and emergent surgery occurred during the procedure.

**Table 2:** Procedural data

Procedure data (*N* = 6)	
Success deployment n, (%)	6 (100%)
LV perforation n, (%)	0 (0%)
Operative approach	
Femoral artery n, (%)	6 (100%)
Device Size	
PVR 65/65 s n, (%)	0 (0%)
PVR 75/75 s n, (%)	3 (50%)
PVR 85/85 s n, (%)	2 (33.3%)
PVR 95/95 s n, (%)	1 (16.7%)
Device related adverse events (*N* = 6)	
Unsuccessful delivery n, (%)	0 (0%)
Unsuccessful deployment n, (%)	0 (0%)
Vascular complications n, (%)	0 (0%)
Acute thrombosis n, (%)	0 (0%)
Emergent surgery n, (%)	0 (0%)

*Abbreviation*: *PVR* percutaneous ventricular restoration

The procedure of the device implantation is briefly illustrated in Fig. 2. The PARACHUTE device was expanded and implanted in the LV apical wall. LV angiography was performed once again to evaluate the geometry of LV (Fig. 2e and f). As displayed in Fig. 2, LV volume was remarkably reduced in the systole and diastole phases.

### Clinical and functional outcomes

**Fig. 3:**
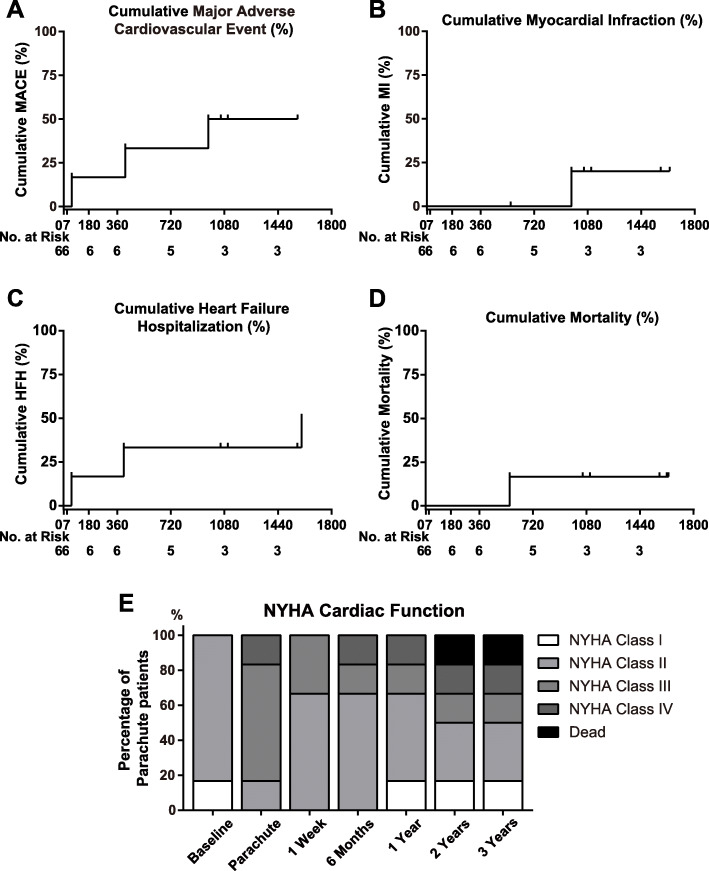
Cumulative incidence of cardiovascular events in PARACHUTE-implanted patients. **a** Cumulative event rates for the composite of cardiovascular death, myocardial infarction, stroke, hospitalization for unstable angina, coronary revascularization, or heart failure end-point. **b**, **c**, and **d** Cumulative myocardial infarction, hospitalization, and mortality, respectively. **e** Changes in functional capacity according to NYHA. MACEs, major adverse cardiovascular events; MI, myocardial infarction; HFH, hospitalization for heart failure. NYHA, New York Heart Association class

Patients in our center were followed up for 4.6 ± 1.7 years. Safety end-points are illustrated in Table 3 and Fig. 3. MACEs occurred in three (50%) patients (Fig. 3a). The most frequently reported MACEs were HF and unplanned interventional therapy. Three subjects (50%) received coronary artery intervention; one of them was diagnosed with non-ST elevation MI, other two have progressive angina attack. Three patients developed HF on the 85th, 418th, and 1599th days. on the 568th day due to progressively worsening HF. The device-related MI and emergent/selective cardiac or aortic surgery were not observed (Table 3).

**Table 3:** Major adverse cardiac events and medication therapy

Major Adverse Cardiac Events	
All-cause mortality n, (%)	1 (16.7%)
Myocardial infarction n, (%)	1 (16.7%)
Emergent cardiac or aorta surgery n, (%)	0 (0%)
Selective cardiac or aorta surgery n, (%)	0 (0%)
Unplanned interventional therapy n, (%)	3 (50%)
New or worsening HF	3 (50%)
Erosion of device through LV	0 (0%)
Cardiac tamponade	0 (0%)
Device migration	0 (0%)
Device embolization	1 (0%)
Peripheral embolization/stroke	0 (0%)
Medication Therapy	
DAPT n, (%)	6 (100%)
Statin n, (%)	6 (100%)
Warfarin n, (%)	6 (100%)
β-Blocker n, (%)	6 (100%)
ACEI/ARB n, (%)	5 (83.3%)
Spironolactone n, (%)	5 (83.3%)

*Abbreviations*: *ACEI* angiotensin-converting enzyme inhibitors, *ARB* Angiotensin receptor blocker;

Our center observed the successful delivery of the PARACHUTE device through 6 months follow-up without device-related MACEs. The medication therapy of the subjects was consistent from inclusion into the trial up to one-year follow-up. DAPT, Statin, Warfarin, and β-blocker were 100% taken in all patients; ACEI/ARB and spironolactone were 83.3%. Even though all patients received warfarin for more than 12 months, device-related embolization was found in a patient by CT and TTE.

Our center analyzed echocardiographic parameters of these subjects at baseline and each time of follow-up, including the LVEF, LVEDD, and estimated PAP. We collected the baseline data after the first attack of anterior MI. Subsequently, data were obtained before the PARACHUTE device implantation and 1 week, 6 months, 1 ± 0.25 year, 2 ± 0.5 years, and 3 ± 0.5 years after device implantation.

**Fig. 4:**
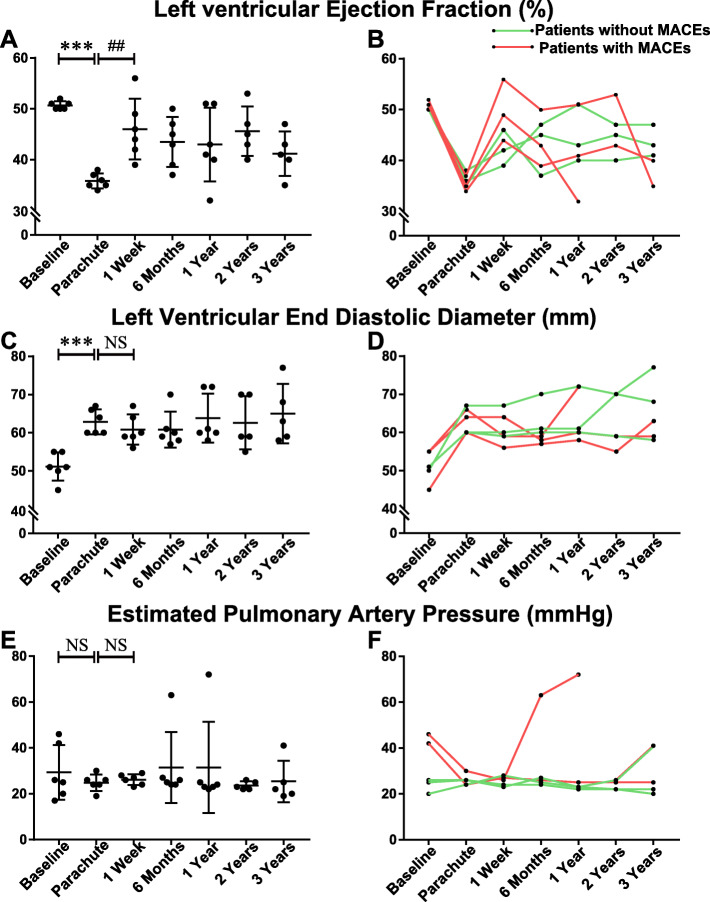
Transthoracic echocardiograph-based cardiac function assessment. Transthoracic echocardiography (TTE)-based cardiac function evaluation was performed at baseline (the first time for MI treatment); before the PARACHUTE device procedure; and at 1 week, 6 months, 1 year, 2 years, and 3 years after device implantation. **a**, **c**, and **e** Comparison of each time point for the left ventricular ejection fraction, left ventricular end-diastolic diameter, and estimated pulmonary artery pressure assessment, respectively. **b**, **d**, and **f** Changes in the TTE-based parameter measurement mentioned above. ***: *P <* 0.001, compared with baseline; ##: *P <* 0.01, compared with the PARACHUTE procedure; NS: not significant. mm: millimeter

We further performed a per- verse post-implantation pairwise comparison of echocardiography. The results of TTE-based cardiac function assessment are shown in Fig. 4. Compared with the baseline, the LVEF was significantly reduced at the time when patients enrolled in the trial (50.67 ± 0.82% vs. 35.83 ± 1.47%, *P <* 0.001). PARACHUTE remarkably elevated LV systolic function in 1 week (35.8 ± 1.47% vs. 46.00 ± 6.0%, *P* = 0.009, illustrated in Fig. 4a and b). For the subjects without MACEs, compared with the one-week measurement, the LVEF demonstrated relatively sustained improvement when we referred to the latest follow-up (42.33 ± 3.51% vs. 43.67 ± 3.06%, *P* = 0.757). By contrast, patients with MACEs often showed a moderate decline (49.67 ± 6.03% vs. 35.67 ± 4.04%, *P* = 0.112).

Patients showed a significant chronic increase in LVEDD before the procedure (51.17 ± 3.71% vs.62.83 ± 3.25%, *P <* 0.001). After the implantation, TTE exhibited no pronounced change in LVEDD, and the progressive LV enlargement was inhibited (Fig. 4c and d). We further measured estimated PAP in Fig. 4e and f, except for the early death of one patient due to HF. The estimated PAP was comparable in each time evaluation.

**Fig. 5:**
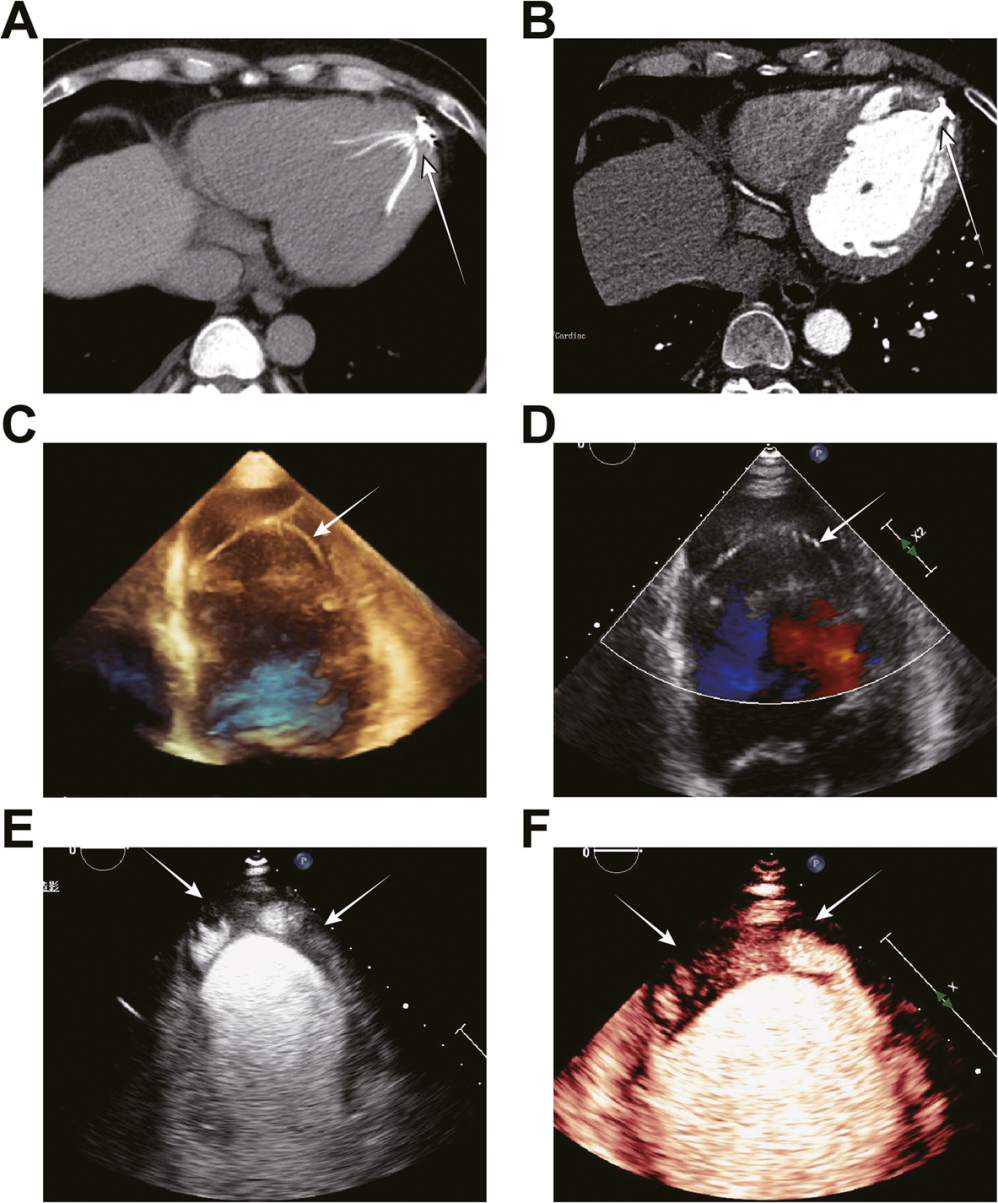
Case with left ventricular thrombus after device implantation by contrast echocardiography. **a** CT scan right after the PARACHUTE implantation to check potential device-related migration or embolization. **b** Latest follow-up detected by contrast-enhanced CT. The filling defect appeared in the apical left ventricle. **c** and **d** 3D-TTE and 2D-TEE were applied to exclude device migration, respectively. The white arrow indicates the implanted device. **e** and **f** Left ventricular thrombus confirmed by LVO and MCE, respectively. The white arrow points to the filling defect-implied LV apical thrombus

Notably, the device-related MACEs also contributed to the poor prognosis. Here, we found one case of embolization that may worsen the state of illness (Fig. 5). Figure 5a is an image captured immediately after PARACHUTE implantation. The latest contrast-enhanced CT of the LV (Fig. 5b) showed the occurrence of device-associated embolization. We conducted a 3D TTE and Doppler scan to exclude device migration (Fig. 5c and d). Furthermore, MCE and LVO were employed to confirm the thrombus located in the LV apex (Fig. 5e and f). These results suggested that the patients should continue to receive warfarin to prevent stroke, even though no embolization-associated adverse events are found. Moreover, long-term anticoagulant treatment may help prevent thrombus-related events.

## Discussion

In this study, we demonstrated that PARACHUTE therapy could be beneficial for the maladaptive remodeling of patients with post-MI by restoring LV ejection and suppressing continuous and overwhelmed LV enlargement, thereby improving patients’ long-term prognosis especially in LVEF-sustained preserved subjects. Limited by the size of cohort, the conclusion may not be robust, however, it could be an alternative treatment for specific group of anterior myocardial infarction patients.

Typical LV dilation is common in patients with an anterior infarction [6]. As a consequence of anterior MI, the LV continuously develops geometrically from ellipsoid to spherical [7], accompanied with chamber dilation and wall motion abnormalities. The dilation of LV increased ventricle volume elevates filling pressure, resulting in the enhanced burden of remaining myocytes, progressive subendocardial myocardial ischemia [8], hemodynamic disorder such as functional mitral regurgitation [4], HF, and death [9]. Many studies have proven that LV remodeling is an independent clinical predictor of prognosis in patients with HF [10]. The beneficial effects of drugs or medical devices on LV remodeling have also been associated with reduced long-term mortality [11]. Therefore, effective and safe treatment strategies are urgently needed to restore LV function and improve outcomes of patients with HF.

To exert the maximum benefit effect to HF patients in this trial, restricted heart features were carefully evaluated, false tendons and ventricular thrombosis are the contraindications of the PARACHUTE procedure. Six eligible subjects were enrolled to the study, the cautiously HF patient selection also contribute to procedural safety eventually. The aforementioned studies [4, 12–14] suggested that the PARACHUTE device can reduce cardiac dimensions and end-diastolic wall stress and improve cardiac output. However, the three-year results indicated a reduction of LVEF and stroke volume. The PARACHUTE IV trial was terminated due to death or rehospitalization for worsening HF.

The device restored enrolled patients’ LV systolic function, and this result was consistent with the findings of prior studies [4, 5, 13]. LVEF significantly improved from preimplantation to 7 days after deployment (35.83 ± 1.47% vs. 46.00 ± 6.00%, *P* = 0.009). The LVEDD and estimated PAP were relatively stable, which suggested that this therapy was effective and safe for patients. The improvement in NYHA functional class was also observed in other patients (illustrated in Fig. 3).

Patients with prior MI have a much higher risk to develop HF and multivessel coronary artery disease than their counterparts [15]. The device is designed to prevent maladaptive LV remodeling, which is helpless in inhibiting recurrent coronary attack. Therefore, personalized treatment should be sought to decrease coronary risks. The HF that occurred in half of our enrolled patients may be partly because they selected suffered from severe cardiac remodeling, thereby enhancing the ratio of cardiac adverse events. However, the device-related MACEs also contributed to the poor prognosis.

As this clinical trial previously designed, all patients received low-dose aspirin and anticoagulation with warfarin for at least 12 months of post-device implantation. The duration of antiplatelet or anticoagulation is based on the spontaneous reendothelialization time. Extra anticoagulation is unnecessary when the implants are completely reendothelialized. The concept is also applicable to the PARACHUTE device. In color doppler flow imaging, we observed that blood flow crossed the device, suggesting it was not entirely reendothelialized even after 3 years, so thrombosis may be promoted. The evidence implies that the PARACHUTE device may need to be refined to fit the geometry of the dilated ventricle.

Despite the small number of patients in our center, we have the longest clinical observation of the PARACHUTE cohort in China. In this trial, the efficiency and safety of the PARACHUTE procedure were investigated, and long-term MACE and cardiac function were evaluated. These data add further evidence to PARACHUTE studies and prove that the ventricular partition device is relatively safe and may reduce long-term death. However, the PARACHUTE device could increase the HF ratio.

### Limitations

The study was limited by its small sample size; unblinded, single-arm nature; and single-center clinical observations. Given that it was conducted in self-control mode and lacked a control group using standardized medical therapy or SVR, we could not rule out potential bias in the adjudication process. Furthermore, a robust conclusion could not be made without control group efficacy.

## Conclusions

The PARACHUTE device is an alternative therapy for patients with severe LV maladaptive remodeling. However, the PARACHUTE device seems to increase the HF ratio at 4.6 years follow-up.

## Supplementary Information

**Additional file 1: Supplementary Figure 1.** Mandatory use of screening cardiac CT to assure the appropriate inclusion of patients. Anatomical insights into patients are provided to allow mechanistic interpretation of the device performance. (A) Measurement of the height from the apex to the implant landing zone. (B) Extra cardiac function assessment on the basis of TTE evaluation. (C) and (D) Diastolic and systolic measurement parameters, respectively.

## Data Availability

All data generated or analysed during this study are included in this published article.

## Abbreviations

HF: Heart failure
LV: Left ventricular
LVEDD: LV end-diastolic diameter
ICDs: Implantable cardioverter defibrillator
CRT-Ds: Cardiac resynchronization therapy
SVR: Surgical ventricular reconstruction
NYHA: New York Heart Association
TTE: Transthoracic echocardiography
MCE: Myocardial contrast echocardiography
LVO: LV opacification
MACEs: Major adverse cardiac events
PCI: Percutaneous coronary intervention
CABG: Coronary artery bypass grafting
PVR: Percutaneous ventricular restoration
ACEI: Angiotensin-converting enzyme inhibitors
ARB: Angiotensin receptor blocker
