# Supplementary figures and images for: Long-term prognosis analysis of PARACHUTE device implantation in patients with ischemic heart failure: a single-center experience of Chinese patients
Source: J Cardiothorac Surg. 2021 Apr 20;16:98. doi: 10.1186/s13019-021-01484-0 (PMC8056655; doi:10.1186/s13019-021-01484-0)

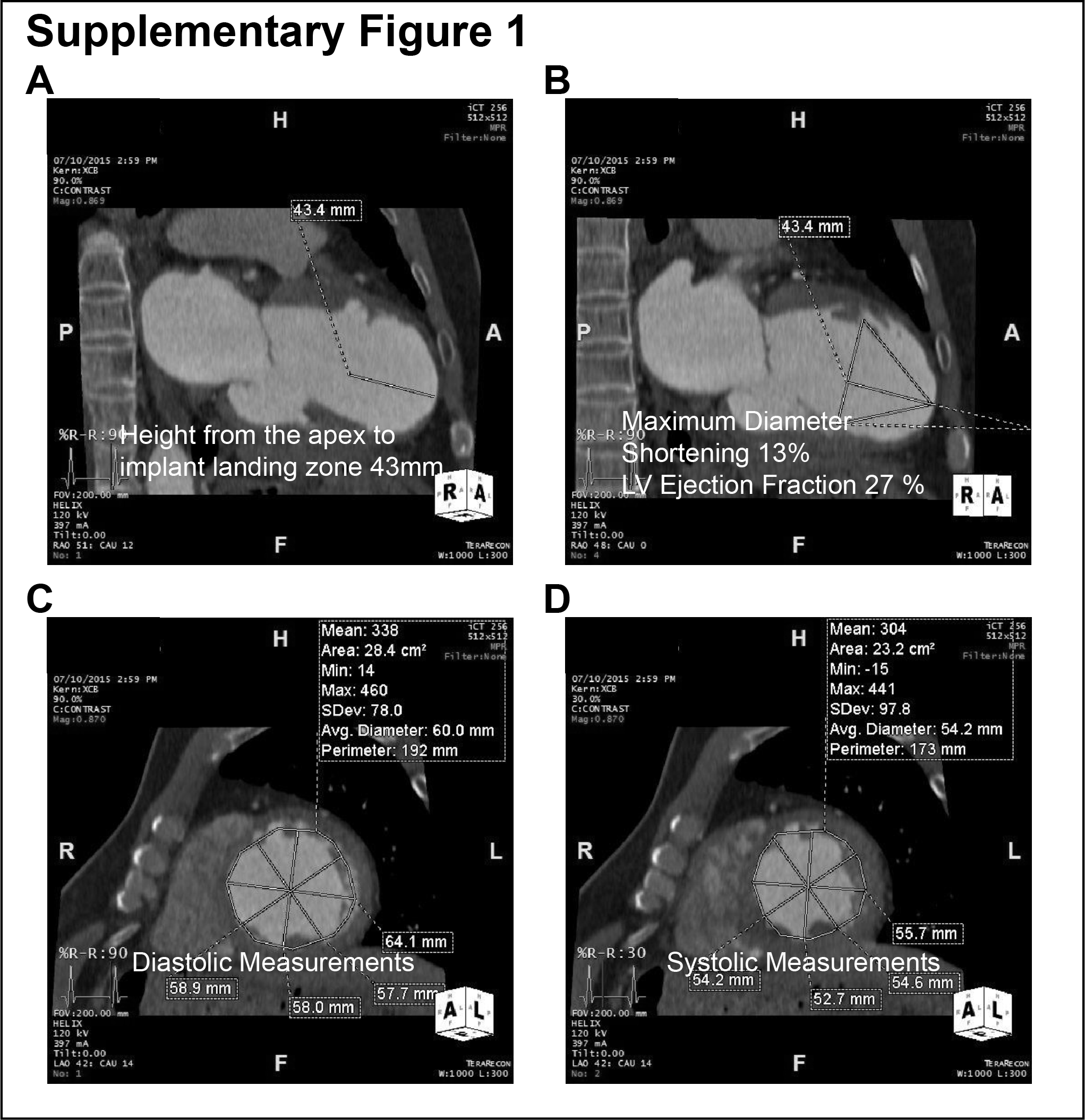

Supplement: Supplementary file 1 — Additional file 1: Supplementary Figure 1. Mandatory use of screening cardiac CT to assure the appropriate inclusion of patients. Anatomical insights into patients are provided to allow mechanistic interpretation of the device performance. (A) Measurement of the height from the apex to the implant landing zone. (B) Extra cardiac function assessment on the basis of TTE evaluation. (C) and (D) Diastolic and systolic measurement parameters, respectively. [file 13019_2021_1484_MOESM1_ESM.tif]
